# The Mentoring Circuit: An Innovative Speed‐Dating Approach to Support Career Awareness in Speech and Language Therapy

**DOI:** 10.1111/tct.70077

**Published:** 2025-03-17

**Authors:** Claudia Kate Au‐Yeung, Yi‐Ting Chia, Andrea Fernando, Serena Lo

**Affiliations:** ^1^ Warwick Medical School Coventry UK; ^2^ Speech Therapists' Links UK; ^3^ KK Women's and Children's Hospital Singapore Singapore; ^4^ Central London Community Healthcare NHS Trust London UK

**Keywords:** speech and language therapy, slt, mentoring circuit, speech therapy, mentoring

## Abstract

**Background:**

Traditional placements in speech and language therapy (SLT) courses pose challenges for students to gain exposure and exchange knowledge within a wide range of specialties in the profession. To counteract this, we piloted an innovative ‘mentoring circuit’ to provide an online opportunity for student and newly qualified SLTs to gain insights and network.

**Approach:**

Mentees and mentors were recruited through professional networks and snowball sampling methods. Six working SLTs from different specialties volunteered as ‘mentors’ and were allocated individual breakout rooms on Zoom. Students, ‘mentees’, were grouped according to SLT stages, were given the opportunity to ask questions to each specialist in 10‐min intervals. Each group of mentees were rotated along the circuit until they met all six specialists. Feedback was obtained via an online questionnaire.

**Evaluation:**

Fifteen mentees (67% students, 33% newly qualified SLTs) attended. Key themes emerged from mentees' feedback, including the value of learning from a diverse range of specialists, benefits of peer learning and optimal mentees–mentor ratio. Suggestions included additional time for follow‐up questions. Mentors also reported positive experiences, expressing enjoyment in sharing knowledge, networking opportunities, opportunities to reflect and appreciation for the circuit's format. They suggested additional time per breakout room and receiving questions in advance.

**Implications:**

Feedback showed that the mentoring circuit effectively provided insights and facilitated the sharing of clinical practices among participants. Both mentees and mentors reported positive experiences, indicating a mutually beneficial mentoring relationship. This approach can be considered by other healthcare professions to support clinical placement learning during the students' degree.

## 1 Background

Speech and language therapy (SLT) encompasses a wide range of specialties from neonatal feeding difficulties and developmental communication difficulties to neurogenic dysphagia and communication disorders. SLTs also work in various settings, from mainstream and special schools to acute and community hospitals. SLT courses equip students with foundational knowledge required for managing communication and swallowing difficulties through lectures and workshops, as well as clinical placements that provide opportunities for students to apply their theoretical knowledge into practice and learn key professional and clinical skills [1]. However, student SLTs often face limitations in gaining sufficient insights and experience across different specialties due to factors such as programme variability, placement and clinical educator availabilities, geographical constraints and students' preferences [2]. This can impact students' job selection and readiness upon graduation [3].

Inspired by speed dating, we piloted an online, innovative approach named the ‘mentoring circuit’ to offer a ‘taster’ session for students and newly qualified therapists (NQTs) to address the challenges of limited exposure to diverse specialties in SLT. Similar to speech dating, the mentoring circuit foster meaningful exchanges by allowing participants to ask SLT specialists questions in small groups, making it a practical solution in time‐pressured NHS educational environment. This approach has been shown to be effective in providing career guidance and boost professional confidence and networking among other early‐stage healthcare professionals, such as surgical trainees [4], medical students [5] and scientists [6] within a time‐efficient framework. Our goal was to allow participants to gain insights into different specialties, inform potential clinical interests and offer guidance on career training pathways where they would not have otherwise been exposed to during their studies or placements, all within a short period of time. The study therefore aims to explore (i) the participants' perceptions of the effectiveness of this approach in raising awareness of different specialties and (ii) how it influenced participants' immediate impact on confidence, knowledge, and career exploration experience.

Inspired by speed‐dating, we piloted an online, innovative approach named the ‘mentoring circuit’ to offer a ‘taster’ session for students and newly‐qualified therapists (NQTs) to address the challenges of limited exposure to diverse specialties in SLT. … Similar to speech‐dating, the mentoring circuit foster meaningful exchanges by allowing participants to ask SLT specialists questions in small groups, making it a practical solution in time‐pressured NHS educational environment.

## 2 Approach

The circuit was conducted nationally in the United Kingdom on a Saturday in August 2021 via Zoom. For the purpose of this article, student SLTs and NQTs are known as ‘mentees’, and SLT specialists are known as ‘mentors’.

### 2.1 Recruitment

Mentees and mentors were recruited via professional networks and snowball sampling methods across the United Kingdom. Professional networks involved Royal College of Speech and Language Therapy (RCSLT), university SLT societies, and professional Twitter accounts. All participants signed up voluntarily. All mentors were registered under the Health & Care Professions Council.

### 2.2 Mentoring Circuit Delivery

**FIGURE 1:**
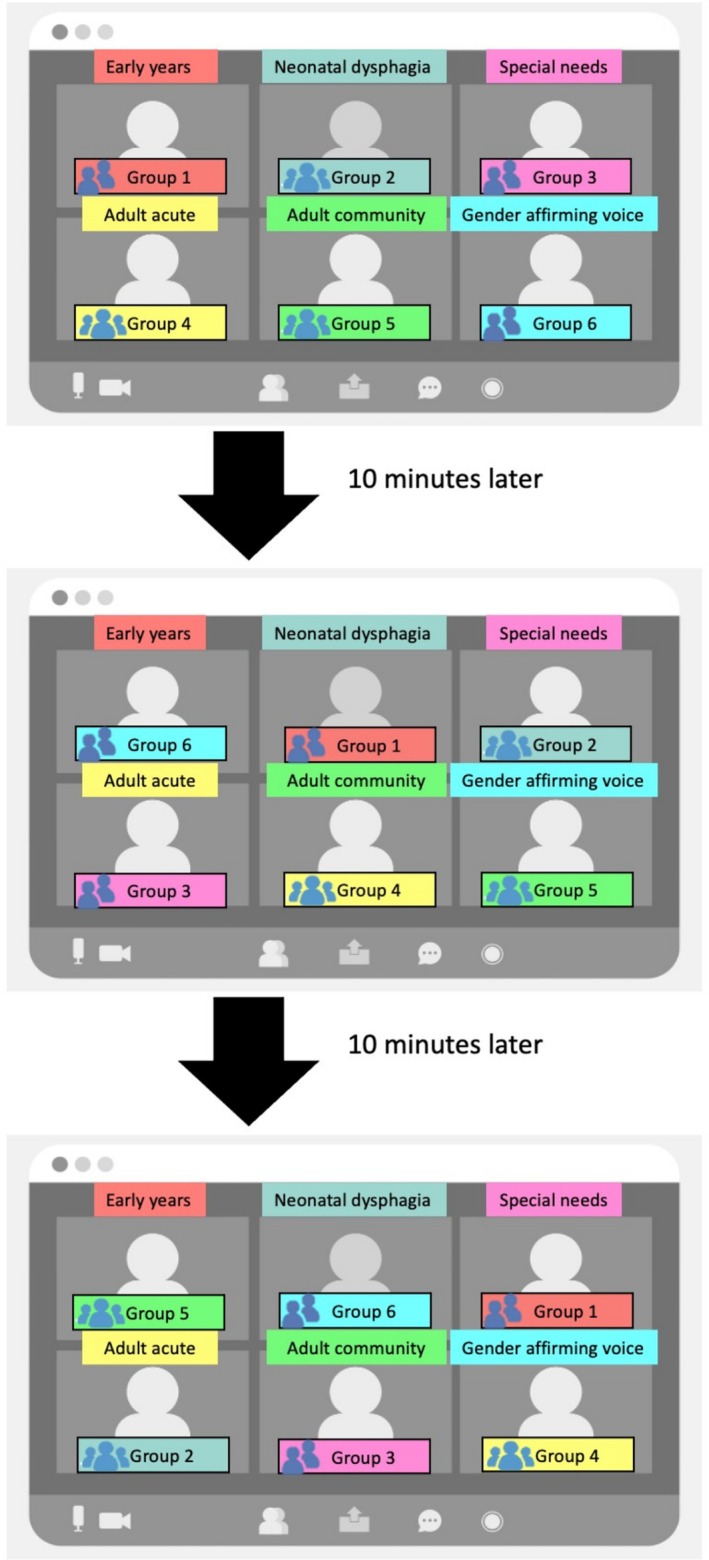
Specialist SLTs, mentors, were each allocated to a Zoom breakout room. They subspecialized in early years, neonatal dysphagia, special needs, adult acute, adult community and gender affirming voice. Two to three mentees were grouped and assigned to a mentor to ask any questions. After 10 min, the groups were moved along the ‘circuit’ to meet the next mentor. This process was repeated six times in total until the mentees met all six mentors.

Six working SLTs from varying specialities volunteered as mentors. They worked in adult acute and community services, mainstream and special needs school, neonatal dysphagia and transgender voice services. Each specialist was assigned a breakout room. Two to three mentees were grouped according to SLT stages (i.e., students and NQTs) to ensure career‐relevant and engaging environment. Mentees rotated among the specialist rooms every 10 min and had the opportunity to ask questions. Mentees were encouraged to ask open‐ended questions and focus on topics such as, clinical day‐to‐day responsibilities, challenges and rewards of the specialty and advice for preparing for clinical roles. See Supporting Information for suggested questions provided to mentees. They were rotated along the circuit until each mentee group had met all six specialists. Mentors were briefed to create an open and welcoming space by initiating conversations with general introductions and inviting questions by sharing their own career journeys, reflections and advice based on mentees' stages. See Figure 1 for a visual representation of the mentoring circuit methodology. Feedback was gathered from all participants anonymously after the event.

### 2.3 Feedback Design

Feedback was collected via two online Google Form surveys using Likert scales and free‐text responses. Questions were designed to maximise time efficiency and response rate.

#### 2.3.1 Mentee Questionnaire

Mentees shared their career stage, personal clinical experience and feedback via two closed statements with Likert scales (1—*strongly disagree*, 5—*strongly agree*) and four open‐ended questions with free‐text responses.

#### 2.3.2 Mentor Questionnaire

Mentors were asked to answer (i) 2 hybrid questions via checklists and free‐text boxes, (ii) 5 closed statements with a 5‐point Likert scale and (iii) 5 open‐ended questions via free‐text responses.

Quantitative data were analysed using descriptive statistics. Qualitative data were analysed using content analysis identified by three researchers to reduce subjective bias [7]. The whole data set was reviewed twice in a data familiarisation exercise (C.K.A.Y., Y.T.C. and A.F.). All data were line‐by‐line coded independently to categorise free‐text responses into positive and area of improvement, and within so, further identifying and quantifying recurrent themes. This was initially completed independently to reduce subjective bias and maximise inter‐rater reliability. The identified themes were then reviewed, compared and discussed until mutual agreement was reached. As there were no substantial disagreements, re‐coding was not carried out.

## 3 Evaluation

### 3.1 Participant Characteristics

A total of 24 mentees signed up, with 15 mentees participating in the mentoring circuit. Mentees consisted of 67% (*n* = 10) students and 33% (*n* = 5) NQTs from seven institutions in the United Kingdom.

### 3.2 Mentees' Feedback

All mentees (100%) reported that the circuit provided insights into working as a specialist SLT. Eighty‐seven per cent (*n* = 13) of mentees strongly agreed and agreed that the circuit has increased their confidence and knowledge, whereas 13% (*n* = 2) remained neutral.

Three positive themes emerged from mentees' feedback: (i) learning from a diverse range of specialists (*n* = 12, 80%), (ii) peer learning (*n* = 3, 20%) and (iii) structure of the circuit involving 3 mentees: 1 mentor (*n* = 2, 13%). One area of improvement identified was to allow additional time to ask follow‐up questions (*n* = 12, 80%). See Table 1 for feedback and exemplar quotes.

**TABLE 1:** Mentees' feedback post‐event regarding what they enjoyed and what can be changed about the mentoring circuit.

Theme	Representative exemplary quotes
Learning from diversity of specialties	I liked that I could get a quick fire overview of different specialties and have an opportunity to know more about the various areas of specialisation that I could go into as a newly qualified therapist and after my newly qualified years The opportunity to talk to SLTs in specialist areas that you may not come across, for example, on student placement I really enjoyed getting to know different SLTs working in different fields and also getting to know more about areas that are not really taught (or emphasised much) at university I loved the varied panel of speech therapists, where I got to hear about fields I had not even considered before
Peer learning	It was also really helpful to hear from other mentees about where they are in their journey and learn from questions they asked I really liked being in each room with other mentees as they asked questions which I had not thought of!
Structure of the mentoring circuit	Hearing about their SLT journey overall and being able to ask very specific questions within small groups. The number of people per group was appropriate for the time we had (3 mentees) The circuit was also well organised, which allowed me to hear from each speaker without spending too much time on one speaker I would have liked more time in our breakout room to ask questions, I think 10 min is a bit too short. Maybe if the event was longer we could spend 15–20 min in a room instead?

### 3.3 Mentors' Feedback

All mentors (*n* = 6, 100%) strongly agreed that they enjoyed sharing with the mentees and were motivated to participate in the mentoring circuit again. Four themes were identified from mentors' free‐text responses, which include (i) positive experience in sharing specialist knowledge and advice (*n* = 5, 83%), (ii) networking with other specialists and fledgling SLTs (*n* = 3, 50%), (iii) opportunities to reflect on own experiences and journeys (*n* = 2, 33%) and (iv) format of the circuit, including 3 mentees: 1 mentor and its innovative approach (*n* = 2, 33%). Areas for improvement identified were receiving questions ahead of time (*n* = 2, 33%) and having additional time per room (*n* = 2, 33%). See Table 2 for feedback and exemplar quotes. They shared the memorable questions asked by mentees (Table 3), which were consistent with the topics encouraged.

**TABLE 2:** Mentors' feedback post‐event regarding what they enjoyed and what can be changed about the mentoring circuit.

Themes	Example
Sharing specialist knowledge and advice	Always fun to meet soon‐to‐be therapists and chat about the career—makes you feel all positive and motivated again! I really loved sharing my experiences and advice with students and newly‐qualified therapists. Everyone had great questions and was super keen to know more
Networking (meeting students and newly‐qualified therapists)	It was a great opportunity to meet students/newly qualified therapists and also hear about other trusts/services through the students (placements) and other specialists I liked seeing students' enthusiasm!
Opportunities to reflect on own experiences and journeys	I realised that I have not been reflecting much on my experiences recently; interacting with the participants prompted me to look back at my experiences and reflect more in depth
Format of the circuit	The groups of 3 worked really well—gave just about enough time for everyone to ask questions (would not want groups any bigger than 3) It might be good to gather some questions beforehand that we can have a think about answers for to save some time on the day. Slightly longer slots would be good also. It often took a little while for the mentees to ask their questions

**TABLE 3:** Mentors' feedback on the most memorable question asked by mentees.

Adult SLTs	What's the difference between ENT and trans voice work? What was your favourite dysphagia case?
Paediatric SLTs	The NHS is very stretched, and I feel that the degree does not necessarily prepare you for this, how does this impact you? How do you manage? What is the most fulfilling part about working with neonatal population?

## 4 Implications

We piloted an accessible and innovative online‐learning approach inspired by speed dating—the mentoring circuit, which aims to allow students and NQTs to gain insights to a range of paediatric and adult SLT specialties, inform potential clinical interests and offer guidance on career training pathways. Our feedback showed that the mentoring circuit, with its rotational approach, was effective in mentees gaining insights into different clinical specialties in a single session. Although the primary aim of the mentoring circuit was for the mentees' benefit, mentors' positive experiences and feedback revealed a mutually beneficial relationship.

Mentees' feedback suggested that they valued this new method of learning to gain a brief overview of specialties and inspire interest to guide further learning and career exploration. Furthermore, mentees complimented the delivery of the circuit, including its structure, mentee groupings and range of specialist mentors available. Mentees were grouped into small groups according to SLT stages to facilitate better engagement and allow more targeted advice for different participant groups. Surprisingly, mentees highlighted that this also supported peer learning via modelling and sharing insights. For instance, one mentee shared, ‘I liked hearing the other mentees' questions especially since we are all at different stages’. These results showed that the mentoring circuit that grouped unfamiliar mentees of similar career stages facilitated peer learning and promoted collaborative engagement among participants, allowing mentees to build on one another's enquires to explore range of topics. This is further reflected by a mentees' feedback, highlighting ‘I also really liked being in each room with other mentees as they asked questions which I hadn't thought of!’ and ‘I feel motivated to continue learning about the speech and language therapy profession’. However, such an experience may not be replicable as it relies on individual participants and group dynamics that flourish within the event.

Surprisingly, mentees highlighted that this also supported peer learning via modelling and sharing insights. For instance, one mentee shared, ‘I liked hearing the other mentees' questions especially since we are all at different stages’.

Our study demonstrated that similar networking and professional development events can be implemented online. This allowed participants across the country to participate and exchange clinical knowledge from various hospital trusts and universities. Traditionally, in‐person conferences provide these opportunities but may not be aimed at students and NQTs. Online implementation allowed for inclusivity, reduced travelling costs and maximised accessibility for both mentees and mentors from diverse backgrounds [8, 9]. This inclusivity played a crucial role in the effectiveness of the mentoring circuit, as it brought together participants with diverse experiences and perspectives, fostering a rich learning environment. This design principle can be valuable in other allied healthcare professionals with similar challenges exploring specialties including nursing, occupational and physiotherapy.

This inclusivity played a crucial role in the effectiveness of the mentoring circuit, as it brought together participants with diverse experiences and perspectives, fostering a rich learning environment.

The ad hoc nature of the event encouraged participation of both mentors and mentees. This, combined with the minimal preparation work, made it a feasible option for mentors working in clinical settings. This is particularly crucial when placement educators, like our mentors, were under pressure to prioritise clinical duties within the NHS [2]. The circuit was designed to require minimal preparation from mentors to reduce time burden, with questions and answers based on personal knowledge and experiences. However, we acknowledge that mentors preferred to receive potential questions in advance. This may help to prepare and consolidate responses within the short time interval. Despite mentors devoting time outside of working hours, 100% mentors reported a positive experience, highlighting opportunities to reflect and share, and network with aspiring and fledgling SLTs. These results suggest the benefits outweigh time burden.

Despite mentors devoting time outside of working hours, 100% mentors reported a positive experience, highlighting opportunities to reflect and share, and network with aspiring and fledgling SLTs.

Moreover, mentees may have different priorities, schedules and learning objectives. Consequently, some may prefer short‐term rather than long‐term mentoring relationships; group rather than individual mentoring; and/or having generalised rather than personalised questions answered [10]. As mentioned, this approach was inspired by speed dating where people of similar interests can be inspired with minimal energy, effort and commitment [4]. The mentoring circuit designed for SLTs therefore allows the mentees to gain ‘a quick overview’ that facilitates preparation for first job interviews and future career plans without long‐term commitments.

All participants reflected a desire to have longer discussion time per breakout room. We therefore plan to increase the time intervals from 10 to 20 min per room. We also aim to gather and offer a list of questions to mentors in advance for future circuits. Future studies can evaluate whether these strategies can enhance mentees and mentors' experiences.

The mentoring circuit was not designed to replace the traditional clinical placements, but to serve as a complementary approach to support clinical learning and provide exposure to a wide range of specialties for students to navigate their career pathways. We acknowledge that this is a small sample study and has a potential of participation bias, as mentees who signed up were likely to be proactive and interested in specialist roles. It should also be highlighted that the mentoring circuit was an initial pilot study designed for students and NQTs within the SLT profession, limiting its generalisability. However, these results provide a starting point for understanding the feasibility, structure and immediate impact on career exploration, peer‐learning and networking opportunities. Generalisability to other healthcare professions, therapists at different career stages, international practices and long‐term impacts should be explored in future studies. Potential collaborations with professional organisations, such as RCSLT, may facilitate larger groups of specialists or international participants to offer networking and learning opportunities in exchanging international practices.

In conclusion, the mentoring circuit is an accessible and innovative method for introducing SLT specialties to students and NQTs. It offered valuable specialist insights, peer learning opportunities and professional development. The positive experiences reported by both mentees and mentors highlight the potential benefits of this approach in facilitating networking, career exploration and knowledge exchange within the SLT community.

## Author Contributions

**Claudia Kate Au‐Yeung:** conceptualization, methodology, investigation, writing – original draft, review, editing, formal analysis, supervision. **Yi‐Ting Chia:** methodology, investigation, writing – original draft, review, editing, formal analysis. **Andrea Fernando:** methodology, investigation, analysis, writing – review and editing. **Serena Lo:** methodology, investigation, analysis, writing – review.

## Ethics Statement

This study involves human participants. NHS Research Ethics Committees tool was consulted and confirmed that ethical approval was exempted. This study is based on voluntary willingness to sign up and provide feedback anonymously. There was no potential harm to participants, and no participants were identified from the collected feedback. All participants accepted consented to the use of data for publication purposes when registering to feedback collection.

## Conflicts of Interest

The authors declare no conflicts of interest.

## Supporting information


**Data S1** Supporting Information.

## Data Availability

The data that support the findings of this study are available from the corresponding author upon reasonable request.
