# Supplementary material for: The Mentoring Circuit: An Innovative Speed‐Dating Approach to Support Career Awareness in Speech and Language Therapy
Source: Clin Teach. 2025 Mar 17;22(3):e70077. doi: 10.1111/tct.70077 (PMC11913764; doi:10.1111/tct.70077)
Supplement: Supplementary file 1 — Data S1 Supporting Information. [file TCT-22-e70077-s001.pdf]

Make the most out of it!

Here are some suggested questions that you can ask. Don't be shy to ask for any personal advice and questions!

1. What is the journey like going into your specialty?
2. What is the most challenging thing you have overcome so far?
3. What would you tell your student/NQT self? What is one thing you wish you knew?
4. What does your day to day job look like?
5. How do you manage your time between research, clinical, and teaching duties?
6. What is in your NQT bag?
7. What do you think makes a good speech therapist?
8. What would you consider your greatest achievement so far?
9. What do you enjoy the most in this area?
10. Have you always wanted to go into this specialty? Why?
11. How did you get into this area?
12. What is your advice for people who might also want to go into this specialist area?
13. What are some challenges you have in your day to day job?
14. Do you have a most memorable patient and what did you learn from him/her?
15. Who inspires you? Why?
16. What is the most commonly used therapy activity you use with your clients?
17. What is a common question that comes up in interviews within your field?

Top tip: Ask open ended questions!
